# Can Mobile Phone Apps Influence People’s Health Behavior Change? An Evidence Review

**DOI:** 10.2196/jmir.5692

**Published:** 2016-11-02

**Authors:** Jing Zhao, Becky Freeman, Mu Li

**Affiliations:** ^1^ School of Public Health Sydney Medical School The University of Sydney Sydney Australia

**Keywords:** review, mobile phone apps, apps, behavior change, intervention, mHealth

## Abstract

**Background:**

Globally, mobile phones have achieved wide reach at an unprecedented rate, and mobile phone apps have become increasingly prevalent among users. The number of health-related apps that were published on the two leading platforms (iOS and Android) reached more than 100,000 in 2014. However, there is a lack of synthesized evidence regarding the effectiveness of mobile phone apps in changing people’s health-related behaviors.

**Objective:**

The aim was to examine the effectiveness of mobile phone apps in achieving health-related behavior change in a broader range of interventions and the quality of the reported studies.

**Methods:**

We conducted a comprehensive bibliographic search of articles on health behavior change using mobile phone apps in peer-reviewed journals published between January 1, 2010 and June 1, 2015. Databases searched included Medline, PreMedline, PsycINFO, Embase, Health Technology Assessment, Education Resource Information Center (ERIC), and Cumulative Index to Nursing and Allied Health Literature (CINAHL). Articles published in the *Journal of Medical Internet Research* during that same period were hand-searched on the journal’s website. Behavior change mechanisms were coded and analyzed. The quality of each included study was assessed by the Cochrane Risk of Bias Assessment Tool.

**Results:**

A total of 23 articles met the inclusion criteria, arranged under 11 themes according to their target behaviors. All studies were conducted in high-income countries. Of these, 17 studies reported statistically significant effects in the direction of targeted behavior change; 19 studies included in this analysis had a 65% or greater retention rate in the intervention group (range 60%-100%); 6 studies reported using behavior change theories with the theory of planned behavior being the most commonly used (in 3 studies). Self-monitoring was the most common behavior change technique applied (in 12 studies). The studies suggest that some features improve the effectiveness of apps, such as less time consumption, user-friendly design, real-time feedback, individualized elements, detailed information, and health professional involvement. All studies were assessed as having some risk of bias.

**Conclusions:**

Our results provide a snapshot of the current evidence of effectiveness for a range of health-related apps. Large sample, high-quality, adequately powered, randomized controlled trials are required. In light of the bias evident in the included studies, better reporting of health-related app interventions is also required. The widespread adoption of mobile phones highlights a significant opportunity to impact health behaviors globally, particularly in low- and middle-income countries.

## Introduction

Globally, mobile phone apps have become increasingly prevalent among users. By July 2015, Google Play, the largest app store, had 1.6 million apps accessible for users. remains the second-largest app store, with 1.5 million apps available for download [1]. There has been a surge of health-related mobile phone apps in recent years. The number of health-related apps released on the two leading platforms, iPhone operating system (iOS) and Android, had reached more than 100,000 in 2014 [2]. Traditionally, health care has been delivered through face-to-face interaction with clinicians. With this new technology at patients’ and health care professionals’ (HCPs) fingertips, people are changing the way they interact. Apps used in health care settings have a number of functions, such as information and time management, communications and consulting, patient management and monitoring, health record maintenance and access, reference and information gathering, and clinical decision making [3]. Although several issues challenge the integration of apps into health care settings (eg, app design is primarily driven by commercial developers), their use has been widely expanded into clinical practice [4,5].

In 2014, the World Health Organization reported that noncommunicable diseases (NCDs) are the leading cause of death globally, responsible for 38 million (68%) of the world’s 56 million deaths in 2012. More than 40% of these deaths (16 million) were premature and avoidable [6]. Simple interventions that decrease NCD risk factors could reduce premature deaths by one-half to two-thirds [7]. Many of these risk factors, such as tobacco use, unhealthy diet, physical inactivity, stress, depression, harmful use of alcohol, overweight, and obesity, can be modified by behavioral change interventions [6]. Apps appear to be an ideal platform to deliver both simple and effective interventions.

In addition to NCDs, health-related apps have the added potential to aid a wide range of target audiences in a whole range of health issues [8]. For example, they can improve contraceptive knowledge of women [9] or help users to prevent nonspecific low back pain [10]. There are also apps designed as intervention tools to encourage healthy habits, such as a sun protection app that provides real-time sun safety advice [11]. Due to the possible positive implications for public health, there is an increasing interest from commercial companies, government agencies, public health organizations, and the general public to utilize apps as a tool for health behavioral change [12-14].

Several reviews have examined the evidence of effectiveness of health-related apps when targeting one specific behavior, such as physical activity, or a specific condition, such as chronic pain [15-19]. Another study reviewed behavioral functionality of apps in health interventions without assessing the quality of the included studies [20]. The aims of this review are to examine the effectiveness of mobile phone apps in achieving health-related behavior change across a broader range of health issues and to examine the quality of the reported studies.

## Methods

### Search Strategy

We searched titles, abstracts, and keywords of peer-reviewed articles published from January 1, 2010 to June 1, 2015. A comprehensive bibliographic search was conducted through Medline, PreMedline, PsycINFO, Embase, Health Technology Assessment, Education Resource Information Center (ERIC), and Cumulative Index to Nursing and Allied Health Literature (CINAHL) by using key search terms, such as mobile application, mobile app, smartphone, and information technology, and using the qualifier “behavior change” (see Multimedia Appendix 1 for the full search strategy). In addition, the *Journal of Medical Internet Research* (*JMIR*) was hand-searched for the same period on the journal’s website.

### Study Selection

We included articles if they were published in English, in a peer-reviewed journal, after 2010, targeted at an adult population, and presented results from the analysis of primary or secondary outcomes. We only included randomized controlled trials (RCTs), case-control studies, and cohort studies that were designed for app-based interventions to improve any health-related behaviors. The exclusion criteria were quasi-experimental studies or qualitative studies; text message, Web, email, Twitter, social network services, or personal digital assistant-based health interventions; absence of behavior change indicators or outcomes; an app was not the primary intervention tool; and articles focused mostly on app design and development. Conference abstracts, protocol papers, reviews, editorials, and commentary were also excluded.

**Figure 1:**
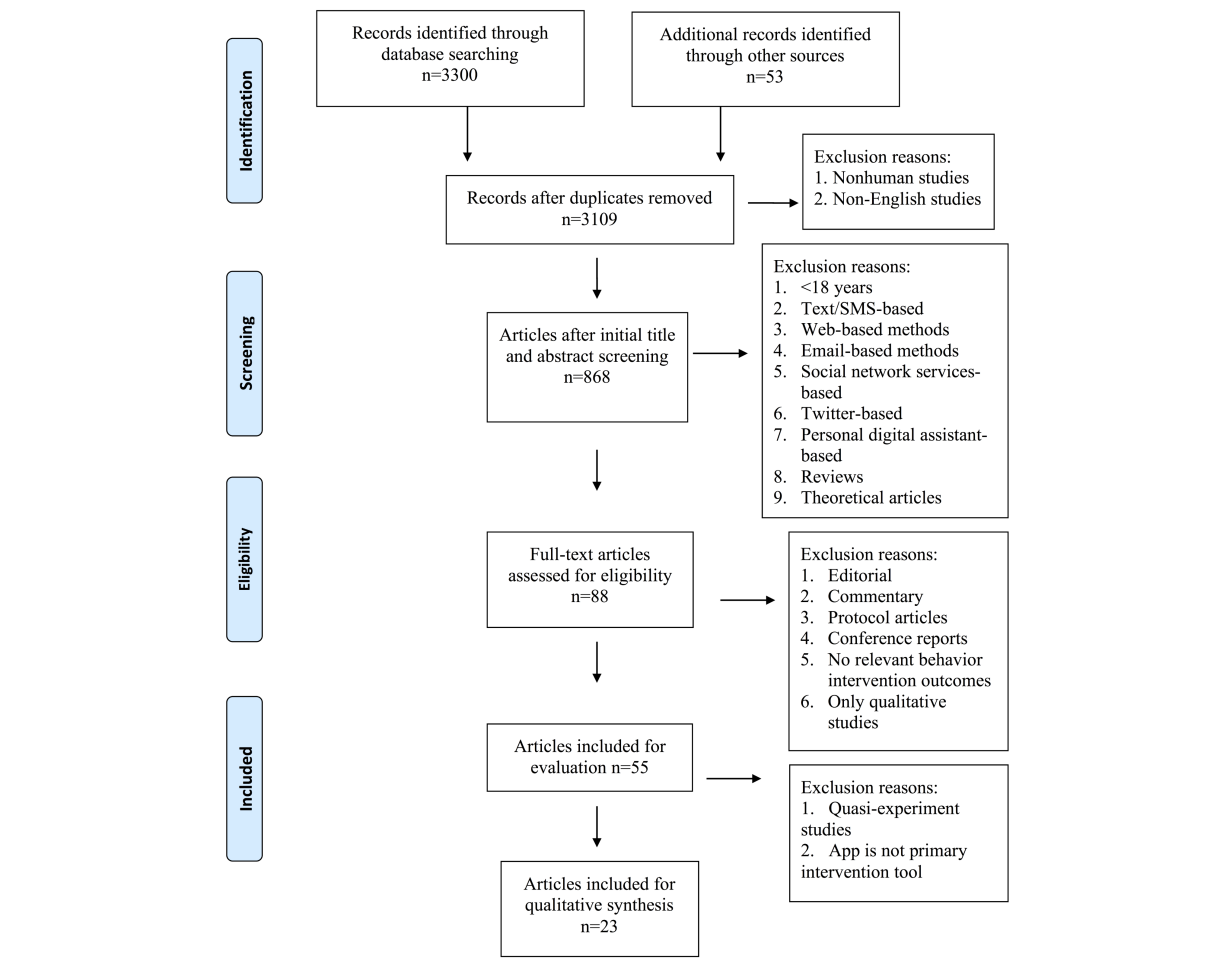
PRISMA 2009 flow diagram.

The initial search returned 3353 articles: 1405 in Medline, 356 in Embase, 791 in CINAHL, 344 in PsycINFO, 296 in ERIC, 71 in PreMedline, 37 in Health Technology Assessment, and 53 in *JMIR*. Following the Preferred Reporting Items for Systematic Reviews and Meta-Analyses (PRISMA) guidelines (Figure 1), we eliminated duplicates and screened the titles and abstracts, which narrowed the results to 868 articles. A full-text review reduced the sample to 88 articles; after applying the exclusion criteria, we further narrowed that to 55 articles, of which 32 were quasi-experiment studies or an app was not the primary intervention tool and they were subsequently excluded. This left a final sample of 23 articles to be included for the review. Studies excluded during the full-text review stage and their reasons for exclusion are listed in Multimedia Appendix 2. Data extraction from identified articles was completed by authors JZ and ML with disagreements resolved through discussion with author BF.

### Data Collection and Analysis

The following information was extracted and analyzed from each of the 23 articles: authors, research location and year of publication, study type, sample size, intervention duration, intervention tools with behavior change mechanisms, target behavior change, control group variables, measurement of behavior change indicators, and reported outcomes and significance levels. The search was kept wide with no specific target health behaviors in the search strategy. Based on the health behaviors identified, the articles were organized into 11 themes: mental health improvement or alcohol addiction, physical activity, weight control and diet control, medication management, lifestyle improvement, diabetes management, sun protection, hypertension management, cardiac rehabilitation, smoking cessation, family planning, and pain management. Apps were deemed effective if they reported quantitative measures of successful behavior change [21]. The characteristics of the studies meeting inclusion criteria are summarized in Multimedia Appendix 3. For trial sample size, large samples usually meant at least 100 participants in each randomized group, moderate sample size was between 60 and 100 participants in each group, and small sample size was less than 60 participants in each group [22,23]. According to the *Cochrane Handbook for Systematic Reviews of Interventions* [22], studies with retention over 80% are classified as having low attrition and studies with retention between 60% and 79% are classified as having moderate attrition. Influencing factors of completing app trials were evaluated to understand determinants of retention rates; features of effective apps were also examined.

Behavior change mechanisms, including the use of theory, techniques, and therapies, were extracted from each study. Behavior change theories applied by the included studies were noted [24]. Behavior change techniques used in the interventions were coded according to Abraham and Michie’s taxonomy of behavior change techniques (BCTs) [25]. Mental health or alcohol addiction apps were most likely to be based on a specific behavior therapy (see Multimedia Appendix 3).

### Study Quality Assessment

All included studies were appraised using the Cochrane Risk of Bias Assessment Tool [22]. This requires assessing each study against a set of seven criteria: random sequence generation, allocation concealment, blinding of participants, blinding of outcome assessment, incomplete outcome data, selective reporting, and other bias. Low risk of bias for completeness of follow-up was defined by a cut-off of 80% complete follow-up [22] (see Multimedia Appendix 4).

## Results

### Characteristics of Included Studies

The 23 articles analyzed in this review were organized under 11 themes according to target behaviors. Of these, 7 targeted mental health or alcohol addiction; 4 targeted increasing physical activity, weight control, and diet control; 3 aimed to improve medication management; 2 involved an intervention for lifestyle improvement; and 1 study was identified in each of the following themes: diabetes management, sun protection, hypertension management, cardiac rehabilitation, smoking cessation, family planning, and pain management. All studies were conducted in high-income countries, 10 in the United States, 3 in Australia, 2 in the United Kingdom and Sweden, respectively, and 1 each in South Korea, Italy, New Zealand, Spain, Switzerland, and the Netherlands. As defined by the inclusion criteria, all included studies used RCT design, except one case-control study [26]. There were 6 large sample studies [10,11,27-30]. A three-arm RCT study had the largest sample size (N=1932) [28], whereas 14 studies had a small sample size (ie, <60 participants per group) [9,26,31-42]. Others had moderate sample sizes. The intervention duration ranged between 3 weeks [36] and 8 months [27]. Of all the apps, only 6 studies evaluated commercially available apps [10,11,29,30,40,41] and 1 study tested a publicly downloadable app developed by the Swedish government [28]; other apps were not publicly available. Only one app, from Switzerland, was designed for people older than age 65 years [40]. All apps were designed in the English language, with the exception of one Spanish app [38]. In total,19 included in this analysis had more than 65% retention in the intervention group with a high of 100% [31,35,36] and a low of 60% [32]. Three studies did not report retention rate [26,34,37] (see Multimedia Appendix 3).

### Mechanisms of Behavior Change

Across the 23 studies, 3 mechanisms were employed to promote behavior change: behavior change theories, BCTs, and specific behavioral therapies. In total, 6 studies reported using behavior change theories to underpin their app interventions [9,10,27-29,36]. The most commonly used theory was the theory of planned behavior [9,10,28], followed by social cognitive theory [29,36]. The top 3 most commonly used BCTs were self-monitoring (12 interventions) [10,27-29,38- 45], feedback provided on performance (8 interventions) [11,28,29,36,37,41- 43], and tailoring messages (8 interventions) [10,26,30,36,38,41- 43]. Apps related to mental health or alcohol addiction were usually based on a specific behavioral therapy, such as motivational enhancement therapy [35], behavioral activation therapy [33], and cognitive behavior therapy [34] (see Multimedia Appendix 3).

**Figure 2:**
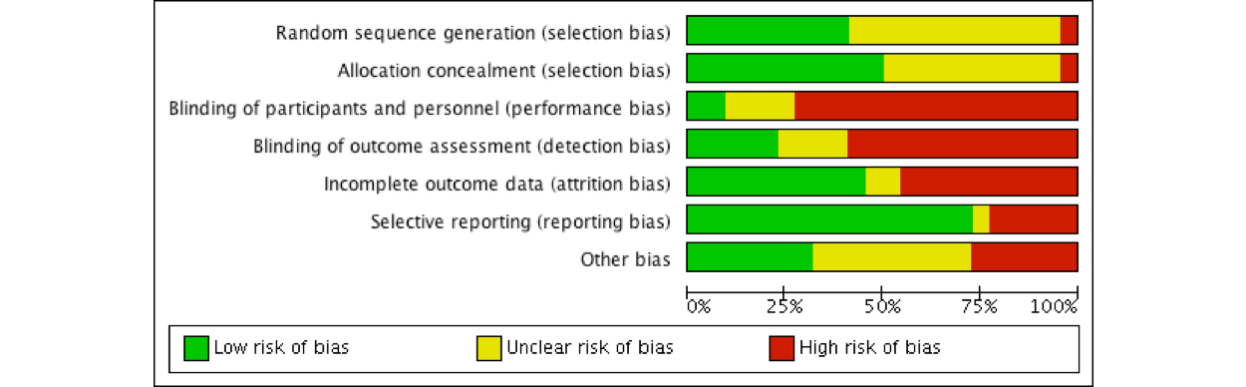
Cochrane risk of bias summary for health behavior change trials.

### Quality of Selected Studies

The quality of reviewed studies is summarized in Multimedia Appendix 4. All 23 studies had some kind of risk of bias according to the Cochrane Risk of Bias Assessment Tool. Only 9 articles adequately reported random sequence generation. A computer random number generator was used in 2 studies [9,27]. The process of minimization, used to make small groups similar, was described in 3 studies [30,43,45]. A total of 11 studies explicitly stated that allocation was concealed (eg, using sequentially numbered opaque, sealed envelopes, central allocation) [9,27-29,31-33,41-44]. Participants were blinded in 1 study, but the assessors had full knowledge of the assignments [36]. Only 1 RCT study of a smoking concession app was double-blinded to the 196 participants and assessors [45]. Assessors were blinded in another 4 studies [9,28,35,38]. Due to the nature of using apps, subject blinding was often not possible across the interventions. The remaining studies were either not blinded or information was not explicitly provided in the reporting. We used a cut-off of 80% completion for low risk of bias for completeness of follow-up [22]. A total of 10 studies were at low risk of attrition bias [9-11,31,35,36,38,42,43,45]. Only 3 studies did not outline the statistical analyses or dropout rate [26,34,37]. With regard to bias of selective outcome reporting, insufficient information was present in 1 study [36] and a high risk of bias was present in 5 studies [30,37,38,40,44]. The quality assessment of the reviewed studies is presented in Multimedia Appendix 4. The Cochrane risk of bias summary is reported in Figure 2.

### Effectiveness of Apps and Features

#### Mental Health or Alcohol Addiction

A total of 7 studies reported on app interventions focused on mental health or alcohol addiction outcomes. Of these, 2 studies described 2 different apps [32,33] that targeted at developing coping skills for different degrees of depression. Watts et al [32] tested the effectiveness of an app delivering a cognitive behavior therapy-based program. There was a statistically significantly improvement on a depression test scale in both the app and computer intervention groups at posttest, and no difference between the 2 groups over time in follow-up. In the other RCT study of a behavioral activation app addressing mild-to-moderate and major depression conducted by Ly et al [33], it was found that the treatment worked significantly better for participants with a more severe form of depression. Ainsworth et al [31] reported that for patients with serious mental illness there was no significant difference in quantitative feedback questionnaire scores, which was developed to assess the acceptability and feasibility between app and text message intervention groups, but there was significant improvement in the app group in 2 other measurements (less time to complete assessment and greater number of data points completed). In a study of a stress management app intervention delivered by oncology nurses, Villani et al [34] found there was a significant decrease in anxiety and significant improvement in affective change in terms of anxiety trait reduction and coping skill acquisition in the intervention group.

In total, 3 RCT studies aimed to lower alcohol consumption among adults. Gonzalez et al [35] demonstrated that an app based on motivational enhancement theory resulted in a significant increase in the percentage of days abstinent among participants with alcohol use disorder over the 6-week study period when compared to controls. In the Gustafson et al [27] study, significantly fewer risky drinking days were achieved in self-determination theory-based app intervention group than the patients in control group. Gajecki et al [28] showed that an app based on theory of planned behavior did not seem to affect alcohol consumption among university students.

#### Increasing Physical Activity, Weight Control, and Diet Control

In total, 4 studies implemented and described app interventions intended to improve physical activity, weight control, and diet control. Rabbi et al [36] found that participants who used an app based on contemporary behavioral science theories walked significantly more than the control group after 3 weeks; further, the users rated the app’s personalized suggestions more positively than the nonpersonalized, generic suggestions created by professionals. Laing et al [29] demonstrated that one of the most popular commercially available weight loss apps, MyFitnessPal, which is based on social cognitive theory, was not effective in helping overweight patients lose weight in a clinical setting over a 6-month period. One case-control study [26] identified significantly decreased weight, fat mass, and body mass index (BMI) in the intervention group compared to controls. Carter et al [43] compared an app intervention group (created on an evidence-based behavioral approach) to two other control groups, one using a paper-based food diary and the other using an online food diary. Over the 6-month study period, adherence to the trial was statistically significantly higher in the mobile phone app group compared with the online website group and the paper diary group. Further, the mean weight change, BMI change, and body fat change were highest in the app intervention group.

#### Medication Management

In total, 3 RCT studies evaluated the effectiveness of apps to improve medication adherence. In an antiretroviral therapy study, Perera et al [37] compared 2 randomized groups using different versions of the same app (an augmented version and standard version) in a 3-month study. There was a significantly higher level of self-reported adherence and decreased viral load among the augmented app group compared to the standard version group. An RCT evaluating an app designed to help elderly Spanish patients reduce nonadherence and medication errors when taking multiple medications reported that app users had significantly better adherence, fewer missed doses, and a significant reduction in medication errors in patients with initial higher rates of errors [38]. In a study of adherence to antidepressant medications among college students, Hammond et al [39] found that there was a strong trend suggesting that the use of a medication reminder app was beneficial in increasing antidepressant medication adherence.

#### Lifestyle Improvement

Only 2 studies measured lifestyle changes in users of 2 commercially available apps. One trial [30] measured changes in health-related behaviors, sleep problems, and fatigue in airline pilots. It found that the intervention arm had a significant improvement in reducing the level of fatigue, improving sleep quality, increasing strenuous physical activity, and changing snacking behavior measures. The other lifestyle study was a three-arm trial to promote walking [40] that included 2 app groups, one using social motivation strategies and the other employing an individual motivation strategy, and a brochure-based control group. The 2 intervention groups both showed significant improvements in total walking time.

#### Other Themes

As shown in Multimedia Appendix 3, only a small number of studies were found under the themes of diabetes management, sun protection, hypertension management, cardiac rehabilitation smoking cessation, family planning, and pain management. Kirwan et al [41] found a freely available app supplemented with text message feedback could significantly improve glycemic control between baseline and 9-month follow-up for patients with type 1 diabetes compared to the control group. One of the first evaluation studies of a commercially available sun protection app [11] showed that only 1/7 sun protection behaviors, wearing wide-brimmed hats, was practiced more by intervention than control participants. In a study comparing an app designed for hypertension management with traditional care [42], the intervention group participants achieved a significant decrease in systolic blood pressure at 12 weeks compared to control participants. Varnfield et al [44] found that the intervention group had significantly higher uptake, adherence, and completion of a cardiac rehabilitation program than the control group. A study of an innovative app addressing heavy smoking showed promising quit rates compared to an app that followed standard US Clinical Practice Guidelines [45]. Gilliam et al [9] noted that young women had a significantly higher knowledge of family planning and increased interest in longer-term contraception methods after using an app-based on the theory of planned behavior. In a three-arm RCT for back pain management [10], users of the app showed significant improvement compared to the control group in every comparison of the critical physical, behavioral, and worksite outcome measures at 4-month follow-up.

#### Suggested Features of Effective App Interventions

Identifying features that enhance intervention effectiveness can inform the development of app-based intervention to produce greater health behavior change and support evaluation of complex interventions. The reviewed studies revealed some important features that could be useful in informing future app intervention design. For example, the MyFitnessPal app incorporates self-monitoring, goal setting, feedback, and social networking features, all deemed critical functions in physical activity and dietary interventions, and it has received the highest possible rating (5/5 stars) from app store reviewers [29]. However, participants in the MyFitnessPal app trial only had minimal change in body weight with no difference between groups. This may be because participants found calorie counting took too much time [29]. This finding is consistent with a previous systematic review suggesting that the amount of participant time required is an important consideration for physical activity and health eating interventions [46].

Another example is that despite receiving no training on how to use the app, the usage of the diabetes management app was high among participants, and there was significantly improved glycemic control in the intervention group between baseline and follow-up at 9 months compared to the control group. This may be attributed to a number of important features of this study, such as the user-friendly design, usefulness of the information, usability of the app, and additional weekly personalized text-message feedback from a health care professional [41]. One important feature of the trial improving airline pilots’ health-related behavior and sleep was the tailored advice, supplemented by additional background information available on the website [30].

## Discussion

In total, 17 studies reviewed reported statistically significant effects in the targeted behavior change, and only one app seemed to have had a negative effect among men with an alcohol use disorder [28]. In one study, behavior change to increase meditation adherence did not reach statistical significance [39]. In total, 10 studies used active comparators that were shown to be also effective; although the intervention groups did not outperform their comparator, the effectiveness of these apps should be considered. For example, in a study to improve patients’ coping skills with depression, mobile phone apps and computer groups were both associated with statistically significant benefits at posttest assessment [32]. Interventions including an active comparator could ensure that all patients who agree to participate in the trial will not be knowingly disadvantaged [47]. Further, this could provide some insight to the app developers for the preferred mode of delivery between apps and existing alternatives, like Web-based or text message-based interventions.

In total, 14 studies had quite small sample sizes, and their findings must be interpreted with caution. Additionally, the long-term sustainability of effects is largely unknown. Trials of larger sample size and longer intervention duration or follow-up time are warranted to assess effectiveness of mobile phone app interventions. The quality of the included studies in terms of high risk of bias in selection, performance, detection, or attrition, and the quality of reporting of the interventions in some of the articles also calls for more rigorous study design and reporting.

With respect to the mechanisms of behavior change, it is important to use theory to inform intervention design as well as specifying BCTs [48,49]. It is apparent that interventions based on behavior change theory are more effective than those lacking a theoretical basis [48-50]. In our review, only 6 studies explicitly reported using behavior change theories to underpin their app interventions [9,10,27-29,36]. In total, 21 studies explicitly reported BCTs were incorporated; the other 2 studies [33,35] did not mention any BCT used in the intervention. However, it seemed that the number of BCTs used did not predict effectiveness. For example, the smoking cessation app study reported that applied five BCTs—self-monitoring, goal setting, self-tracking, social support, and being motivated—did not significantly improve outcomes in smoking cessation compared to the control group [45], whereas the pain management app with three BCTs showed significant improvement compared to the control group in every comparison [10]. In our review, the most commonly adopted BCT (in 12 studies) was self-monitoring, but results were mixed in terms of how effective this technique was in changing behavior. This finding may be a consequence of different BCTs targeting different aspects of the behavior change process.

Retention rate is defined as the proportion of participants who remained in the study to completion. Despite the potential convenience and benefits to app users, only 10 studies in our review achieved a high retention rate (>80%) in intervention group [9-11,31,35,36,38,42,43,45]. The My Meal Mate app [43] is a weight loss intervention with a high retention rate; 40 of 43 (93%) participants returned for follow-up at 6 months. Compared with other similar apps, the key features of the My Meal Mate app are expert-designed, tailored content and weekly supportive text messages. Similarly, the FitBack app had a high retention rate of 92% (183/199) and also tailored content to users’ preferences and interests; participants achieved greater improvement in all physical, behavioral, and worksite outcome measures than the control group [10]. Varnfield et al [44] had a 77% (46/60) completion rate in the home care cardiac rehabilitation app intervention group, which was approximately 30% more than the control group. The involvement of experts who provided weekly scheduled telephone consultations with informed, personalized feedback on progress according to participants’ goals likely contributed to this relatively higher level of participant retention. In a poststudy survey, users rated MyBehavior’s personalized suggestions more positively than the nonpersonalized and generic suggestions [36]. Personalization and adaptation in real time appear to be key elements in engaging a diverse group of participants [51]. This is reinforced by Tang et al [52], who found that young adults highly valued the personalized features of a weight loss app. These studies support that tailored information, real-time feedback, and expert consultation are the app functions that might be most acceptable and useful to participants. In turn, it is likely that these features could result in maintaining higher retention rates and enhancing intervention effectiveness. Further, our findings also indicate that apps with a simple interface and that make better use of app design and technology may reduce the time required for users to participate in the intervention and improve retention. Identifying features that may enhance intervention effectiveness could inform the development of health behavior change apps and support the evaluation of complex interventions.

### Implications for Future Research and Practice

Mobile phone apps are seen as a potential low-cost way to deliver health interventions to both general and at-risk populations. Many such apps exist; however, rigorous research to test their effectiveness and acceptability is lacking. There were 7 publicly available apps that were used in the reviewed studies [10,11,28-30,40,41]. Despite their apparent popularity, public and commercial apps have not been comprehensively evaluated to date; they are currently being used without a thorough understanding of their associated risks and benefits [53]. There is a gap between app concept, delivery, and translation into health behavior change.

The Cochrane Risk of Bias Assessment is a good tool to assess the quality of intervention trials. However, in our findings, the “blinding of participants and personnel” was poor; only one study [45] was double-blinded due to the unique nature of app interventions. The quality of mHealth evidence reporting could be improved through the use of recently published guidelines to aid better understanding and synthesizing findings. The Consolidated Standards for Reporting Trials (CONSORT) provides a 22-item checklist for reporting Web-based and mHealth RCTs [54]. The mHealth Evidence Reporting and Assessment (mERA) checklist could also aid quality improvement of mHealth intervention reporting [55]. Additionally, the Transparent Reporting of Evaluations with Nonrandomized Designs (TREND) statement could assist to improve the reporting quality of nonrandomized evaluations of public health interventions [56]. In this review, only 4 studies described “blinding of outcome assessment” [9,28,35,38]. It might be possible to blind outcome assessors, those doing data analysis, or those administering co-interventions, which is one of the 22 essential items recommended in the CONSORT checklist [54]. It is important for researchers to adopt these guidelines vigilantly for better reporting and communication of research results.

One of the primary benefits of apps is their potential for incredibly high reach. With mobile phone use reaching near saturation among some populations, particularly young adults, and the high rates of consumer acceptability, app effectiveness research must also consider total app reach. This aspect of health behavior change apps has not been assessed, with most studies being exceptionally small in scale. Apps that offer even a small health benefit could still be a valuable public health intervention if the population-level reach is high enough. But, encouragingly, we identified some registered large-scale clinical trial protocols of app-based interventions, suggesting that the current limited scientific evidence may be eased in coming years [57-60].

All identified studies were conducted in high-income countries, which could be partly due to our search criteria limiting publications in English only. However, it is also possible that a significant demand for app research on health behavior change in lower- and middle-income countries is being neglected. The burden of NCDs, such as heart disease, diabetes, cancer, and mental disorders, is high in low- and middle-income countries and is predicted to grow [4]. Mobile phones have great potential to reach populations that previously had restricted access to interventions or health care information [61]. Apps have also created new opportunities and possibilities to reach populations who were largely unreachable via traditional health care channels [62]. mHealth interventions have a positive impact on some chronic diseases in developing countries [63] and text messaging has been recognized as a successful tool to improve behavior change outcomes [13,15]. In comparison with text messaging only, mobile phone apps offer more active engagement in health care and improved convenience at substantially lower cost. However, the current evidence base for the use of app-based interventions in developing countries remains small [64]. The widespread adoption of mobile phones highlights a significant opportunity to impact health behaviors globally, particularly in low- and middle-income countries.

### Limitations

Limitations of this review are worth noting. The search terms are restricted to health behavior change, and we focused mostly on medicine- and health science-related databases, which may have excluded publications in other areas. Although iPhone and Android app stores debuted in June 2007 [65], they have experienced exponential growth in popularity since 2010; some relevant articles published before January 2010 could have been missed. The included studies were all conducted in high-income countries where the health care systems are different from many low- and middle-income countries, which limits the ability to draw generalizable conclusions [66]. The inclusion of studies targeted at the adult population could also confine interpretations about whether app-based interventions can influence behavior change among younger users.

### Conclusions

To our knowledge, no previous study has completed a comprehensive thematic literature review of mobile phone apps for health behavior change. Although a majority of the studies reviewed reported statistically significant effects in targeted behavior change, adequately powered and relatively longer duration RCTs are still required to determine the effectiveness of app-based interventions. Further research should focus on conducting evaluation research in low- and middle-income countries. Moreover, these results highlight the need for better reporting of health-related app interventions. Collaborations between researchers, HCPs, app developers, and policy makers could enhance the process of delivering and testing evidence-based apps to improve health outcomes.

## Appendix

Multimedia Appendix 1 Search strategy.

Multimedia Appendix 2 Studies excluded during full text review.

Multimedia Appendix 3 Characteristics of selected studies.

Multimedia Appendix 4 Study Quality Assessment.

## Abbreviations

BCT: behavior change technique
BMI: body mass Index
CONSORT: Consolidated Standards for Reporting Trials
HCP: health care professional
iOS: iPhone operating system
JMIR: Journal of Medical Internet Research
PRISMA: Preferred Reporting Items for Systematic Reviews and Meta-Analyses
RCT: randomized control trial
NCD: noncommunicable disease
TREND: Transparent Reporting of Evaluations with Nonrandomized Designs
