# Supplementary material for: Can Mobile Phone Apps Influence People’s Health Behavior Change? An Evidence Review
Source: J Med Internet Res. 2016 Nov 2;18(11):e287. doi: 10.2196/jmir.5692 (PMC5295827; doi:10.2196/jmir.5692)
Supplement: Multimedia Appendix 1 [file jmir_v18i11e287_app1.pdf]

Multimedia Appendix 1: Search strategy

**Medline**
(Biomedical technology/or “mobile phone technology”.tw /or ehealth.tw /or mhealth.tw /or “mobile app$1”.tw /or Internet/or "smart phone*".tw/ or “smartphone*”.tw/or “Mobile phone*”.tw/or handheld/software/or “mobile application”.tw/or Cell phones/or computer handheld /or “mobile device*”/or “iphone”.tw/or “ipad”.tw/or Medical informatics application/ “Medical informatics application”.tw /or” Android”.tw/or “tablet computer”.tw) ***and*** (Health behavior/ or patient compliance /or self-examination/ or tobacco use cessation /or treatment refusal/ or /illness behaviour or /“behavio?r change”.tw
Limits: English, humans and year: January 1, 2010 - June 1, 2015
**Embase:**
(“android”.mp. or “mhealth”.mp. or “tablet computer”.mp. or “ipad”.mp. or “ipod” .mp. or “mobile phone”/exp or “internet” /exp or “telehealth”/exp or “smartphone”.mp. or “iphone” .mp.or “mobile papplication” /exp or “medical informatics application” .mp.or “ mobile technology”.mp.) ***and*** “behavior change”/exp and “intervention”.mp.
Limits: English, humans and year: January 1, 2010 - June 1, 2015
**PsycINFO**
(Cellular phones/or cell phones/or mobile phones/mobile devices/or telephone systems/or “mobile phone”.tw/or Biotechnology /or information technology/or “mobile device*”.tw/or Telemedicine/or learning/or mobile application*.tw /or “Medical informatics application*”.tw /or Health care delivery/or health promotion/or healthcare service/ “mhealth”.tw /or Health education/”ehealth”.tw /or “android”.tw /or Online therapy/or smartphone*.tw/or smart phone*.tw /or Internet/or “mobile app$1”.tw/ or “tablet*”.tw /or “personal digital assistant”.tw /or “ipad”.tw/or “iphone”.tw / ***and*** behavior change/ or lifestyle changes/or Behavior Modification/or "behavio?r* change*".tw.
Limited: English, year: January 1, 2010 - June 1, 2015, peer-reviewed journal human
**CINAHL:**
(MH "Behavioral Changes" /or MH "health behavior" /or TI "behavior change" /or AB "behavior change") ***and*** (MH "Mobile Phone"/or MH “Smartphone”/or TI smartphone /or AB smartphone /or MH "Cellular Phone"/or TI "cellular phone"/or AB "cellular phone" /or MH Software/ or TI software/or AB software/ or MH "Wireless Communications" /or TI "wireless communication"/ or AB "wireless communication"/ or MH "Preventive Health Care Methods"/or TI "Preventive Health Care Methods"/ or AB "Preventive Health Care Methods"/ or MH "Self Care Methods"/or AB "Self Care Methods" /or TI "Self Care Methods" /or MH

"Computers, Hand-Held"/or AB "Computers, Hand-Held"/or TI "Computers, Hand-Held" /or MH "Reminder Systems"/ or TI "Reminder Systems"/or AB "Reminder Systems" / or MH "Medical informatics"/ or AB "Medical informatics" / or TI "Medical informatics" / or MH "Information seeking behavior"/ AB "Information seeking behavior" / or TI "Information seeking behavior" / or MH "Health Promotion Methods"/ or AB "Health Promotion Methods"/ or TI "Health Promotion Methods" /or MH "Health education Methods"/ or AB "Health education Methods" / or TI "Health education Methods" / or (MH "Consumer Health Information"/ or AB "Consumer Health Information" / or TI "Consumer Health Information"/ or MH "Information Science" / or AB "Information Science"/ or TI "Information Science" / or MH "Health Care Industry"/ or AB "Health Care Industry" / or TI "Health Care Industry"/ or MH "Health Care Delivery"/ or AB "Health Care Delivery"/ or TI "Health Care Delivery"
Limits: Peer Reviewed; English, year: January 1, 2010 - June 1, 2015
**Eric**
(Handhelds/or ipods/ or mobile devices/ or mp3/ or personal digital assistants/ or Telecommunications/ or Educational Technology/“mobile phone*”.tw /or Electronic Learning/ or Technology Uses in Education/ "mobile device*".tw./ or Internet /or information networks/or smart phone.tw/smartphone.tw/ or Health education/or health promotion/or mhealth.tw/ or Ehealth.tw/ or Information technology/or information technology.tw/ or “tablet*”.tw/ or “ipad”.tw/ or “iphone”.tw/ or “e-learning”.tw/ or “mobility of learning”.tw) ***and*** (Behavior Modification/ or Behavior Change/ “Behavior?r change*”.tw)
Limits: English, humans and year: January 1, 2010 - June 1, 2015
**Pre-Medline**
"smart phone*".tw./or "smartphone*".tw./or "ipad".tw./or "iphone".tw./ or "tablet*".tw./or "mobile application*".tw./or "mobile app$1".tw./or "ehealth".tw./or "mhealth".tw./or "e-learning".tw./or "medical informatics application*".tw./or "health care intervention".tw./or "app$1".tw/ or "behavio?r therapy".tw.**and** ("behavio?r* change*".tw. /or "health behavio?r".tw).
Limits: English, humans and year: January 1, 2010 - June 1, 2015
**Health Technology Assessment**
(Technology.tw/or Computer Communication Networks/ or Telecommunications/ or Information Systems/or Computers, handheld /or health promotion /or software /or “mobile app*”.tw /or delivery of health care /or health personnel /or “health care intervention”.tw) ***and*** (behavior?r* change*.tw)
Limits: English, humans and year: January 1, 2010 - June 1, 2015
