# Supplementary material for: Can Mobile Phone Apps Influence People’s Health Behavior Change? An Evidence Review
Source: J Med Internet Res. 2016 Nov 2;18(11):e287. doi: 10.2196/jmir.5692 (PMC5295827; doi:10.2196/jmir.5692)
Supplement: Multimedia Appendix 2 [file jmir_v18i11e287_app2.pdf]

Multimedia Appendix 2: Studies Excluded During Full Text Review

Excluded articles (n=65)

Summary of reasons:

Inclusion criteria not met:

1. App is not primary intervention tool / Article does not have a primary focus on App (n=11)
2. Targeted population under 18 years old (n=3)

Exclusion criteria met:

1. Quasi-experiment studies (n=21)
2. Protocol (n=10)
3. Only qualitative studies (n=8)
4. No relevant behaviour intervention outcomes (n=7)
5. Editorial (n=2)
6. Commentary (n=2)
7. Conference report (n=1)

List of excluded articles:

(1) App is not primary intervention tool / Article does not have a primary focus on App

1. Turner-McGrievy GB, M.W.; Moore, J.B.; Kaczynski, A.T.; Barr-Anderson, D.J.; Tate, D.F. : Comparison of traditional versus mobile app self-monitoring of dietary intake and physical activity among overweight and obese adults participating in the mhealth weight loss program. 2013.
2. Turner-McGrievy G, Tate D: Tweets, Apps, and Pods: Results of the 6-Month Mobile Pounds Off Digitally (Mobile POD) Randomized Weight-Loss Intervention Among Adults. J Med Internet Res. 2013;13(4):e120.
3. Quinn CC, Shardell MD, Terrin ML, et al.: Cluster-randomized trial of a mobile phone personalized behavioral intervention for blood glucose control. Diabetes Technology and Therapeutics. 2011;15(1):S65-S66.
4. Mattila E, Orsama A-L, Ahtinen A, et al.: Personal health technologies in employee health promotion: Usage activity, usefulness, and health-related outcomes in a 1-year randomized controlled trial. Journal of Medical Internet Research. 2013;15(7).
5. Martin CK, Miller AC, Thomas DM, et al.: Efficacy of SmartLossSM, a Smartphone-Based Weight Loss Intervention: Results from a Randomized Controlled Trial. Obesity. 2015;23(5):935-942.
6. Kirwan M, Duncan MJ, Vandelanotte C, et al.: Using Smartphone Technology to Monitor Physical Activity in the 10,000 Steps Program: A Matched Case–Control Trial. J Med Internet Res. 2012;14(2):e55.

7. Hebden L, Cook A, Ploeg HP, et al.: A mobile health intervention for weight management among young adults: a pilot randomised controlled trial. Journal of Human Nutrition & Dietetics. 2014;27(4):322-332.
8. Fukuoka Y, Gay CL, Joiner KL, et al.: A Novel Diabetes Prevention Intervention Using a Mobile App. A Randomized Controlled Trial With Overweight Adults at Risk. American Journal of Preventive Medicine. 2015.
9. Allen JK, Stephens J, Dennison Himmelfarb CR, et al.: Randomized controlled pilot study testing use of smartphone technology for obesity treatment. Journal of Obesity. 2013;2013:151597.
10. Brindal E, Hendrie G, Freyne J, et al.: Design and pilot results of a mobile phone weight-loss application for women starting a meal replacement programme. J Telemed Telecare. 2013.
11. Mays D, Cremeens J, Usdan S, et al.: The feasibility of assessing alcohol use among college students using wireless mobile devices: Implications for health education and behavioural research. Health Education Journal. 2010;69(3):311-320.

(2) Targeted population under 18 years old

1. Mailuhu AK, Verhagen EA, Van Ochten JM, et al.: The trAPP-study: Cost-effectiveness of an unsupervised e-health supported neuromuscular training program for the treatment of acute ankle sprains in general practice: Design of a randomized controlled trial. BMC Musculoskeletal Disorders. 2015;16(1).
2. Huguet A, Stinson J, MacKay B, et al.: Bringing psychosocial support to headache sufferers using information and communication technology: Lessons learned from asking potential users what they want. Pain Research and Management. 2014;19(1):e1-e8.
3. Glynn LG, Hayes PS, Casey M, et al.: Effectiveness of a smartphone application to promote physical activity in primary care: The SMART MOVE randomised controlled trial. British Journal of General Practice. 2014;64(624):e384-e391.

(3) Quasi-experiment studies

1. Ahtinen A, Mattila E, Välkkynen P, et al.: Mobile mental wellness training for stress management: Feasibility and design implications based on a one-month field study. Journal of Medical Internet Research. 2013;15(7).
2. Ben-Zeev D, Kaiser SM, Brenner CJ, et al.: Development and usability testing of FOCUS: a smartphone system for self-management of schizophrenia. Psychiatric rehabilitation journal. 2013;36(4):289-296.
3. Bond DS, Thomas JG, Raynor HA, et al.: B-MOBILE - A smartphone-based intervention to reduce sedentary time in overweight/obese individuals: A within-subjects experimental trial. PLoS ONE. 2014;9(6).
4. Burns MN, Begale M, Duffecy J, et al.: Harnessing context sensing to develop a mobile intervention for depression. Journal of Medical Internet Research. 2011;13(3):e55.
5. Carter MC, Burley VJ, Nykjaer C, et al.: 'My Meal Mate' (MMM): validation of the diet measures captured on a smartphone application to facilitate weight loss. British Journal of Nutrition. 2013;109(3):539-546.

6. Fukuoka Y, Vittinghoff E, Jong SS, et al.: Innovation to motivation-pilot study of a mobile phone intervention to increase physical activity among sedentary women. Preventive Medicine. 2010;51(3-4):287-289.
7. Hasin DS, Aharonovich E, Greenstein E: HealthCall for the smartphone: technology enhancement of brief intervention in HIV alcohol dependent patients. Addiction Science & Clinical Practice. 2014;9:5.
8. Hensel DJ, Fortenberry JD, Harezlak J, et al.: The feasibility of cell phone based electronic diaries for STI/HIV research. BMC Medical Research Methodology. 2012;12:75.
9. Heo J, Chun M, Lee KY, et al.: Effects of a smartphone application on breast self-examination: a feasibility study. Healthc Inform Res. 2013;19(4):250-260.
10. Kaiser PK, Wang YZ, He YG, et al.: Feasibility of a novel remote daily monitoring system for age-related macular degeneration using mobile handheld devices: results of a pilot study. Retina. 2013;33(9):1863-1870.
11. Kim H-Y, Park H-A, Min YH, et al.: Development of an obesity management ontology based on the nursing process for the mobile-device domain. Journal of Medical Internet Research. 2013;15(6):56-66.
12. Mattila E, Lappalainen R, Parkka J, et al.: Use of a mobile phone diary for observing weight management and related behaviours. Journal of Telemedicine and Telecare. 2010;16(5):260-264.
13. Morris ME, Kathawala Q, Leen TK, et al.: Mobile therapy: case study evaluations of a cell phone application for emotional self-awareness. J Med Internet Res. 2010;12(2):e10.
14. Mundi MS, Lorentz PA, Grothe K, et al.: Feasibility of Smartphone-Based Education Modules and Ecological Momentary Assessment/Intervention in Pre-bariatric Surgery Patients. Obesity Surgery. 2015.
15. Patel S, Jacobus-Kantor L, Marshall L, et al.: Mobilizing your medications: an automated medication reminder application for mobile phones and hypertension medication adherence in a high-risk urban population. Journal of Diabetes Science & Technology. 2013;7(3):630-639.
16. Rizvi SL, Dimeff LA, Skutch J, et al.: A Pilot Study of the DBT Coach: An Interactive Mobile Phone Application for Individuals with Borderline Personality Disorder and Substance Use Disorder. Behavior Therapy. 2011;42(4):589-600.
17. Robinson E, Higgs S, Daley AJ, et al.: Development and feasibility testing of a smart phone based attentive eating intervention. BMC Public Health. 2013;13:639.
18. Thomas JG, Wing RR: Health-e-call, a smartphone-assisted behavioral obesity treatment: Pilot study. Journal of Medical Internet Research 2013;15(4).
19. van Dantzig S, Geleijnse G, van Halteren AT: Toward a persuasive mobile application to reduce sedentary behavior. Personal and Ubiquitous Computing. 2013;17(6):1237-1246.
20. Wayne N, Ritvo P: Smartphone-enabled health coach intervention for people with diabetes from a modest socioeconomic strata community: single-arm longitudinal feasibility study. Journal of medical Internet research. 2014;16(6):e149.
21. EunSeok C, Kim KH, Umpierrez G, et al.: A Feasibility Study to Develop a Diabetes Prevention Program for Young Adults With Prediabetes by Using Digital Platforms and a Handheld Device. Diabetes Educator. 2014;40(5):626-637.

(4) Protocol

1. Zhang J, Song YL, Bai CX: MIOTIC study: A prospective, multicenter, randomized study to evaluate the long-term efficacy of mobile phone-based internet of things in the management of patients with stable COPD. International Journal of COPD. 2013;8:433-438.
2. Verwey R, van der Weegen S, Spreeuwenberg M, et al.: A monitoring and feedback tool embedded in a counselling protocol to increase physical activity of patients with COPD or type 2 diabetes in primary care: study protocol of a three-arm cluster randomised controlled trial. BMC family practice. 2014;15:93.
3. Valdivieso-Lopez E, Flores-Mateo G, Molina-Gomez JD, et al.: Efficacy of a mobile application for smoking cessation in young people: study protocol for a clustered, randomized trial. BMC public health. 2013;13:704.
4. Stuckey MI, Shapiro S, Gill DP, et al.: A lifestyle intervention supported by mobile health technologies to improve the cardiometabolic risk profile of individuals at risk for cardiovascular disease and type 2 diabetes: study rationale and protocol. BMC public health. 2013;13:1051.
5. Recio-Rodriguez JI, Martin-Cantera C, Gonzalez-Viejo N, et al.: Effectiveness of a smartphone application for improving healthy lifestyles, a randomized clinical trial (EVIDENT II): study protocol. BMC public health. 2014;14:254.
6. Perez-Cruzado D, Cuesta-Vargas AI: Improving Adherence Physical Activity with a Smartphone Application Based on Adults with Intellectual Disabilities (APPCOID). BMC public health. 2013;13:1173.
7. Pellegrini CA, Duncan JM, Moller AC, et al.: A smartphone-supported weight loss program: design of the ENGAGED randomized controlled trial. BMC Public Health. 2012;12:1041.
8. Glynn LG, Hayes PS, Casey M, et al.: SMART MOVE - a smartphone-based intervention to promote physical activity in primary care: study protocol for a randomized controlled trial. Trials [Electronic Resource]. 2013;14:157.
9. Fukuoka Y, Komatsu J, Suarez L, et al.: The mPED randomized controlled clinical trial: applying mobile persuasive technologies to increase physical activity in sedentary women protocol. BMC public health. 2011;11(1):933-933.
10. Blodt S, Pach D, Roll S, et al.: Effectiveness of app-based relaxation for patients with chronic low back pain (relaxback) and chronic neck pain (relaxneck): Study protocol for two randomized pragmatic trials. Trials. 2014;15(1).

(5) No revelent behaviour intervention outcomes

1. Varnfield M, Karunanithi MK, Sarela A, et al.: Uptake of a technology-assisted home-care cardiac rehabilitation program. Medical Journal of Australia. 2011;194(4):S15-19.
2. Goh G, Tan NC, Malhotra R, et al.: Short-Term Trajectories of Use of a Caloric-Monitoring Mobile Phone App Among Patients With Type 2 Diabetes Mellitus in a Primary Care Setting. J Med Internet Res. 2015;17(2):e33.
3. Faurholt-Jepsen M, Vinberg M, Christensen EM, et al.: Daily electronic self-monitoring of subjective and objective symptoms in bipolar disorder-the MONARCA trial protocol (MONitoring, treAtment and pRediCtion of bipolAr disorder episodes): A randomised controlled single-blind trial. BMJ Open. 2013;3(7).
4. Eyles H, McLean R, Neal B, et al.: Using mobile technology to support lower-salt food choices for people with cardiovascular disease: protocol for the SaltSwitch randomized controlled trial. BMC Public Health. 2014;14:950.

5. Buman MP, Epstein DR, Baldwin CM, et al.: Design and iterative testing of a sleep/physical activity smartphone nullAPPnull for US veterans. Sleep. 2015;38:A438-A439.
6. Block G, Azar K, Block T, et al.: Development and clinical trial of an eHealth program for pre-diabetics. FASEB Journal. 2014;28(1).
7. Batch BC, Tyson C, Bagwell J, et al.: Weight loss intervention for young adults using mobile technology: Design and rationale of a randomized controlled trial - Cell Phone Intervention for You (CITY). Contemporary Clinical Trials. 2014;37(2):333-341.

(6) Only Qualitative outcome

1. Wills J, Gemen R, Harricharan M, et al.: Integrating behaviour change techniques and digital technology for dietitian support in weight management cases. Obesity Reviews. 2014;15:148.
2. West J, Crookston B, Lister C, et al.: Technology for health: A qualitative study on barriers to using the ipad for diet change. Annals of Nutrition and Metabolism. 2013;63:926-927.
3. Weltman G, Lamon J, Freedy E, et al.: Police department personnel stress resilience training: An institutional case study. Global Advances In Health and Medicine. 2014;3(2):72-79.
4. Spook JE, Paulussen T, Kok G, et al.: Monitoring Dietary Intake and Physical Activity Electronically: Feasibility, Usability, and Ecological Validity of a Mobile-Based Ecological Momentary Assessment Tool. J Med Internet Res. 2013;15(9):e214.
5. Palmier-Claus JE, Rogers A, Ainsworth J, et al.: Integrating mobile-phone based assessment for psychosis into people's everyday lives and clinical care: A qualitative study. BMC Psychiatry. 2013;13.
6. Kane JM, Perlis RH, DiCarlo LA, et al.: First experience with a wireless system incorporating physiologic assessments and direct confirmation of digital tablet ingestions in ambulatory patients with schizophrenia or bipolar disorder. Journal of Clinical Psychiatry. 2013;74(6):e533-540.
7. Ball K, Ong KL, Jackson M, et al.: EMPOWER: The development and pilot-testing of an evidence-based weight loss mobile telephone app. Obesity Research and Clinical Practice. 2014;8:5.
8. Arsand E, Tatara N, Ostengen G, et al.: Mobile phone-based self-management tools for type 2 diabetes: the few touch application. Journal of diabetes science and technology. 2010;4(2):328-336.

(7) Conference report

1. Kirwan M, Vandelanotte C, Duncan M, et al.: Using smartphonestoincrease physical activity: Usability testing of the 10,000 Steps iPhone application. Journal of Science and Medicine in Sport. 2010;13:e28.

(8) Editorial

1. Buhi ER: EDITOR’S CHOICE. Digital Health and AJPH: The Time Has Come! American Journal of Public Health. 2015;105(3):420-420.

2. Anonymous: How to put your smartphone "on call." Applications that run on your cell phone put health and wellness aids just a touch away. Harvard Women's Health Watch. 2010;18(4):2-4.

(9) Commentary

1. Lupton D: Health promotion in the digital era: a critical commentary. Health Promotion International. 2015;30(1):174-183.

2. Fottrell E: Commentary: The emperor's new phone. BMJ (Online). 2015;350.
