# Supplementary material for: Can Mobile Phone Apps Influence People’s Health Behavior Change? An Evidence Review
Source: J Med Internet Res. 2016 Nov 2;18(11):e287. doi: 10.2196/jmir.5692 (PMC5295827; doi:10.2196/jmir.5692)
Supplement: Multimedia Appendix 3 [file jmir_v18i11e287_app3.pdf]

Author /location	Study design	Target behavior change	Intervention tools and behavior change mechanisms	Control tool	Primary measurement	Retention rate	Results
**Mental health or Alcohol addiction**							
Ainsworth, J[31]<br>2013<br>UK	RCT<br>19 days<br><br>N=24<br>Age:18-50yr<br>Participants with schizophrenia	Improving diagnostic real-time assessment score in patients with Serious mental illness	Smartphone app<br><br>1.real-time assessment	Text messages	1.Number of data points<br>2.Time took to complete assessment<br>3.Patients' quantitative feedback	100% (12/12) in both groups	1.Significant greater number of data points in intervention group<br>2. Significantly less time to complete the assessment in app group<br>3. No significant differences in participants' quantitative feedback questionnaire scores about the acceptability and feasibility between the two delivery modalities
Watts, S[32]<br>2013<br>Australia	RCT<br>3 months<br><br>N=35<br>Age:over 18yr with major depression	Developing skills in coping sadness in patients with depression	App- based The Sadness Program<br><br>1.cognitive behavioral therapy<br>2.self-report	Computer-based The Sadness Program	PHQ-9	1. 60% (9/15) in intervention group<br><br>2. 46.7% (14/30) in control group	Both the Mobile and Computer Groups were associated with statistically significantly benefits in the PHQ-9 score at post-test. At 3 months follow up, the reduction seen for both groups remained significant

Ly, K. H[33]<br>2014<br>Sweden	RCT<br>8weeks and 6 months follow up<br><br>N=81<br>Mean age 36yr diagnosed with major depressive disorder	Increasing daily activation in patients with depression	Behavioral activation app<br><br>1.based on behavioral activation therapy	Mindfulness app	PHQ-9 and BDI-II	1. 77.5% (31/40) in intervention group<br><br>2. 70.7% (29/41) in control group	1. The two interventions did not differ significantly from one another<br>2. For participants with higher severity of depression, the treatment based on intervention app was superior<br>3. For participants with lower initial severity, the treatment based on control app worked significantly better
Villani, D[34]<br>2013<br>Italy	RCT<br>4weeks and 3moths follow-up<br><br>N=30<br>Female oncology nurses	Improving coping of stress related anxiety	Mobile app- The stress inoculation training<br><br>1.cognitive behavior therapy<br>2.self-help	Neutral video through mobile phones	1.Anxiety level<br>2. Work components 3. Perceived stress<br>4. Coping skills	Not mentioned	1.Results showed a significant decrease in anxiety among intervention group<br>2. A significant improvement in affective change in terms of anxiety trait reduction and coping skills acquisition

Gustafson, D. H[27]<br>2014<br>United States	RCT<br>8 months intervention,<br>4 months follow-up<br><br>N= 349 adults meet the criteria for DSM-IV alcohol dependence	Reducing alcohol addiction	ACHESS app + usual care<br><br>1.based on self-determination theory<br>2.peer support groups, communication addiction experts<br>3.self-monitoring<br>4.reminders	Usual care	Risky drinking days	1. 77.7% (132/170) in intervention group<br><br>2. 77.6% (139/179) in control group	Intervention group had significantly fewer risky drinking days than patients in the control group
Gajecki, M[28]<br>2014<br>Sweden	3 arms RCT<br>7 weeks<br><br>N=1932<br>Uni students in risky alcohol consumption<br>Mean age: 24.7yr	Reducing problematic alcohol intake	Intervention1.Smartphone App: (Promillekoll)<br><br>1.based on theory of planned behavior<br>2.self-monitoring<br>3.feedback provide<br><br>Intervention2. web-based app (PartyPlanner)	Not receive any intervention or feedback on risky drinking	1.Calculating<br>2. Displaying an individual's eBAC	1. App intervention group: 73.6% (473/643)<br><br>2. Web-based intervention group: 60.9% (388/640)<br><br>3. Control group: 66.4% (500/649)	1.The apps studied using eBAC calculation did not seem to affect alcohol consumption among university students<br>2. One app (Promillekoll) may have led to a negative effect among men
Gonzalez. M[35]<br>2015<br>United States	RCT<br>6 weeks<br><br>N=54<br>Age:18- 45yr with an alcohol use disorder	Decreasing alcohol use disorders	APP: LBMI-A<br><br>1.based on Motivational enhancement therapy	Internet-based motivational intervention plus bibliotherapy	1.Percent days abstinent (PDA)<br>2.Percent heavy drinking days (PHDD)<br>3.Drinks per week (DPW)	1.100% (28/28) in intervention group<br><br>2. 76.9% (20/26) in control group.	1.Multilevel models revealed that the LBMI-A resulted in a significant increase in PDA over the course of the study<br><br>2.Both interventions resulted in significant decreases in PHDD and DPW

**Physical activity, weight control and diet control**							
Rabbi, M[36]<br>2015<br>United States	RCT<br>3weeks<br><br>N=17<br>Age:18-49yr	Increasing physical activity and adopting healthy diet	App: MyBehavior<br><br>1.based on contemporary behavioral science theories: (learning theory, social cognitive theory and the Fogg Behavior Model)<br>2.personalized feedback<br>3. Self-tracking	Non-personalized, generic suggestions created by professionals	1.Food (calories in per meal consumed)<br><br>2.Activity (walking, running or exercise durations per day)	100% (9/9) in both groups	1.MyBehavior users walked significantly more than the control group<br>2. Users rated app's personalized suggestions more positively than the non-personalized, generic suggestions created by professionals
Laing, B. Y[29]<br>2014<br>United States	RCT<br>6months<br><br>N=212<br>Primary care patients with BMI >=25 kg/m2<br>Interested in weight loss	Increasing calorie counts	MyFitnessPal app +usual care<br><br>1.based on social cognitive theory,<br>2.self-monitoring,<br>3.self-reminder<br>4.goal setting,<br>5.feedback.<br>6.social networking	Usual care	Weight loss	1. 74% (78 /105) in intervention group<br><br>2. 79% (85/107) in control group	Weight change was minimal, with no difference between groups.
Lee, W[26]<br>2010<br>Korea	Case-control<br>6weeks<br><br>N=36<br>Mean age:29.5 yr<br><br>All voluntary participants from the obese clinic at the fitness center in Soul	1.Increasing nutrition knowledge<br><br>2.Better Weight control	Mobile-phone based app (SmartDiet)<br><br>1.personalized nutrition information<br>2.self-recording<br>3.goal setting	Normal care	1.Fat mass<br>2.Weight<br>3.BMI	Not mentioned	Fat mass, Weight, Fat mass and BMI decreased significantly in the intervention group, but no significant changes in the control group;

Carter C[43]<br>2013<br>UK	3 arms RCT<br>6months<br><br>N=128<br>overweight volunteers<br>Age: 18-65yr,<br>BMI>27	Increasing physical activity and adopting healthy diet	Smartphone app + text-message feedback<br><br>My Meal Mate (MMM)<br>1.evidence-based behavioral approach<br>2.goal setting,<br>3.self-monitoring<br>4.feedback tailored weekly text messages	Control 1：<br>Paper food diary<br><br>Control 2：<br>Online food diary website	Weight<br>BMI<br>Body fat	1. 93% (40/43) in app group<br><br>2. 55% (19/42) in the website group	1. Adherence was statistically significantly higher in the smartphone group with a mean of 92 days (SD 67) of dietary recording compared with 35 days (SD 44) in the website group and 29 days (SD 39) in the diary group.<br><br>2.Mean weight change, BMI change and body fat change were highest in smartphone group
**Medication management**							
Perera, A. I[37]<br>2014<br>New Zealand	RCT<br>3months<br><br>N=28<br>Adults on ART for at least 6 months	Improving adherence to antiretroviral therapy (ART).	Augmented version of the smartphone app<br><br>1.real-time information provided<br>2. 24 hour timer reminding<br>3.feed-back	Standard version of the smartphone app<br><br>1.Only 24 hour timer reminding	1.Self-reported adherence to ART<br>2.Viral load<br>3. Pharmacy dispensing	Not mentioned	1.Intervention group showed a significantly higher level of self-reported adherence to ART at 3 months and decreased viral load as compared to individuals using the standard version<br>2. No significant differences of pharmacy dispensing between two groups
Mira, J.J[38]<br>2014<br>Spain	RCT<br>3months<br><br>N=99<br>Age:65-90yr<br>Patients taking multiple medications	Reducing non-adherence and medication errors	Pillbox App (ALICE)<br><br>1.medication self-management<br>2.personlized advice<br>3.self-monitoring	Oral and written information on the safe use of their medications	1.Morisky Medication Adherence Scale (MMAS-4)<br>2. Safety medication use	100% (48/48,51/51) in both groups	1. Patients with no experience with information and communication technologies reported significantly better adherence and fewer missed doses<br>2. ALICE only helped to significantly reduce medication errors in patients with an initially higher rate of errors

Hammonds, T[39]<br>2015<br>United States	RCT<br>30days<br><br>N=57 College students<br>Age:18-30yr<br>Prescribed an antidepressant	Increasing adherence to antidepressant medications	A medication reminder app + a manual pill count<br><br>1.self-reminder<br>2.self-monitoring	A manual pill count	1.Medication adherence<br><br>2. The Beck Depression Inventory (BDI) scores	1. 67% (20/30) in the treatment group<br><br>2. 74 % (20/27) participants in the control group	1. There was a strong trend suggesting that the use of a medication reminder app was beneficial for adherence to antidepressant medication regimens.<br>2. The magnitude of change in depression symptoms was not greater in the experimental group
**Lifestyle improvement**							
Van Drongelen, A[30]<br>2014<br>Netherlands	RCT<br>6months<br>N= 502 airline pilots	1.Adopting health-related behavior<br>2.Reducing sleep problems and fatigue	App (MORE Energy)<br><br>1.personal advice<br>2.general information<br>3.reminders	Website with standard information about fatigue.	1.Fatigue<br>2.Sleep quality health-related behavior and health	1.Intervention group:79.3% (199/251)<br><br>2.Control group:76% (191/251)	Intervention group showed a significant improvement on fatigue, sleep quality, strenuous physical activity and snacking behavior
Van het Reve, E[40]<br>2014<br>Switzerland	3 arms RCT<br>12 weeks<br>N=48 older than 65yr	1.Improving gait quality<br><br>2.Improving physical performance	Social: Tablet app (ActiveLifestyle) social group added<br><br>Individual: tablet app(ActiveLifestyle)<br><br>1. provide instruction on how to perform behavior<br>2.self- monitors<br>3.goal setting	A training plan on paper sheets	1.Gait performance under single and dual task conditions<br>2.Dual task costs of walking<br>3.Falls efficacy<br>4.Physical performance (SPPB scores)<br>5.The Falls Efficacy Scale International (FES-I)	1. 78.6% (11/14) in the social group<br><br>2. 84.6% (11/13) in the individual group.<br><br>3. 58.8% (10/17) in control group	1. The individual group was significantly faster in gait speed than the control group<br><br>2. The individual group was faster than the social group though not statistically significant.<br><br>3. Social or individual motivation strategies were equally effective

**Diabetes management**							
Kirwan, M[41]<br>2013<br>Australia	RCT<br>6-month intervention,<br>3 month follow-up<br><br>N=72<br>Mean age: 35.20yr<br><br>Mean year of diagnosed with type 1 diabetes: 18.94 years	Improving glycemic control	Smartphone app (Glucose Buddy) + text-message feedback<br><br>1.self-monitoring<br>2.self-management<br>3.weekly personalized text-message feedback	Usual care	1.HbA1c level<br><br>2.Blood test	1.Intervention group: 69.4% (25/36)<br><br>2.Control group: 77.8% (28/36)	The intervention group significantly improved glycemic control (HbA1c) from baseline to 9-month follow-up, compared to the control group.
**Sun protection**							
Buller, D.B[11]<br>2015<br>United States	RCT<br>3weeks intervention,<br>3weeks and 8weeks follow-up<br><br>N=202 adults	1.Increasing use of sun protection<br><br>2.Reducing time spent outdoors in the midday sun	Smartphone app (Solar Cell)<br><br>1.delivering advice<br>2.feedback<br>3.provide information<br>4.self-efficacy	Not receive any app	1. Percentage of days with the use of sun protection<br>2.Time spent outdoors in the midday sun<br>3.Number of sunburns	1. 92.7% (89/96) in intervention group<br><br>2. 98.1% (104/106) in control group	Intervention group used wide-brimmed hats more at 7 weeks than control participants
**Hypertension management**							
Moore, J. O[42]<br>2014<br>United States	RCT<br>12 weeks<br><br>N=44<br>Adults with uncontrolled essential hypertension	Improving hypertension management	CollaboRhythm app on a tablet<br><br>1.self-track<br>2.personlized feedback<br>3.self-monitoring	Traditional care	1.Medication adherence<br>2.Blood pressure (BP)	1.96.5%(22/23) in intervention group<br><br>2.95.2%(20/21) in control group	1. Intervention subjects achieved significant decrease in systolic BP at 12 weeks than control subjects.<br>2.A greater percentage of intervention subjects achieved goal BP ≤ 130/80 mm Hg<br>3.100% of them achieved goal

							BP ≤ 140/90 mmHg.
**Cardiac rehabilitation**							
Varnfield, M[44]<br>2014<br>Australia	RCT<br>6-week intervention and 6-month self-maintenance period.<br><br>N=120<br>Post-myocardial infarction patients	Improving cardiac rehabilitation use in post-myocardial infarction patients	A smartphone-based home service delivery (apps+sms+telephone consultation)<br><br>1.self-monitoring<br>2.uploading app data to web portal	A traditional, center based program	1. 6-min walking test distance<br><br>2.Depression scores	1.Intervention group: 76.7%(46/60)<br><br>2. Control group: 43.3%(26/60)	1. Intervention group had significantly higher uptake adherence and completion rates than control group.<br>2.Both groups showed significant improvements in 6-minute walk test from baseline to 6 weeks
**Smoking cessation**							
Bricker, J. B[45]<br>2014<br>United States	RCT<br>2months<br><br>N=196<br>At least five cigarettes daily for at least past 12 months	Reducing heavy smoking	An innovative app (SmartQuit)<br><br>1.acceptance and commitment therapy<br>2.self-monitoring<br>3.goal setting<br>4.self-tracking<br>5.social support<br>6.motivated	An app following US Clinical Practice Guidelines (QuitGuide)	1.Participant satisfaction, utilization of the app<br>2.Smoking cessation outcomes	1.Intervention group: 81.6% (80/98)<br><br>2. Control group: 85.7% (84/98)	1. The overall quit rates were 13% in SmartQuit vs. 8% in QuitGuide<br><br>2. Promising quit rates compared to QuitGuide app

**Family planning**							
Gilliam ML[9]<br>2014<br>United States	RCT<br>8months<br><br>N=60 women under 30yr old, median age of 22.5yr	Increasing women's interests in up taking long-acting reversible contraceptive (LARC)	App<br><br>1. based on the theory of planned behavior<br>2.general information<br>3. instruction on how to use	Standard care	1.Expressed interest in discussing a LARC method during the clinic visit.<br>2.Contraceptive knowledge and LARC selection	1.Intervention group: 90.3% (28/31)<br><br>2.Control group:77.4% (24/31)	App users had a significantly higher knowledge of contraceptive effectiveness and increased interest in the implant after the intervention
**Pain management**							
Irvine,A B[10]<br>2015<br>United States	RCT<br>8weeks<br>4months follow-up<br><br>N=597 adults suffered low back pain in previous 3 months	1.Improving self-care for on-going pain<br><br>2.Adopting behaviors to reduce the chance of reoccurrence	Group 1: FitBack app<br><br>1. based on Theory of Planned Behavior<br>2. self-management intervention<br>3. self-monitoring<br>4. tailored information<br><br>Group 2: Alternative Website Control	Usual care	Change in responses to the Modified Oswestry Low Back Pain Disability Questionnaire	1.App intervention group: 92.0% (183/199)<br><br>2.Alternative intervention group:97.5% (194/199)<br><br>3.Control group: 97.5% (194/199)	Users of the FitBack program showed significant improvement compared to the control group in every comparison of the critical physical, behavioral, and worksite outcome measures

ADHD: Attention Deficit Hyperactivity Disorder
BG: blood glucose
BP: blood press
BDI: Beck's Depression Inventory
BDI-II: Beck Depression Inventory-II
Depression Scale -PHQ-9: 9-item Patient Health Questionnaire
eBAC: individual's estimated blood alcohol concentration
EQ5D-3L: EuroQol Five Dimensional [EQ5D-3L] questionnaire

EBI: Eating Behaviors Inventory (EBI)
SPPB: Short Physical Performance Battery
SMS: Short Message Service
SSB: sugar sweetened beverages
SBP: Systolic blood pressure
TBP: theory-based podcast
PA: physical activity
