# Supplementary material for: Can Mobile Phone Apps Influence People’s Health Behavior Change? An Evidence Review
Source: J Med Internet Res. 2016 Nov 2;18(11):e287. doi: 10.2196/jmir.5692 (PMC5295827; doi:10.2196/jmir.5692)
Supplement: Multimedia Appendix 4 [file jmir_v18i11e287_app4.pdf]

Multimedia Appendix 4: Study Quality Assessment

**Trial**	**Random sequence generation**	**Allocation concealment**	**Blinding of participants and personnel**	**Blinding of outcome assessment**	**Incomplete outcome data**	**Selective outcome reporting**	**Other bias**
**Mental health or Alcohol addiction**							
Ainsworth, J[31] 2013	U	L	U	U	L	L	U
Watts, S[32] 2013	L	L	H	H	H	L	H
Ly,K. H[33] 2014	L	L	H	H	H	L	L
Villani, D[34] 2013	U	H	H	H	U	L	U
Gustafson, D. H[27] 2014	L	L	H	H	H	L	U
Gajecki,M [28] 2014	U	L	H	L	H	L	U
Gonzalez, M[35] 2015	U	U	H	L	L	L	H
**Physical activity, weight control and diet control**							
Rabbi, M [36] 2015	U	U	L	H	L	U	U

Multimedia Appendix 4: Study Quality Assessment

Laing,B. Y[29] 2014	L	L	H	H	H	L	L
Carter, C [43] 2013	L	L	H	H	L	L	U
**Medication management**							
Perera,A. I[37] 2014	U	U	U	U	U	H	U
Mira, J.J[38] 2014	U	U	H	L	L	H	U
Hammonds , T[39] 2015	U	U	H	H	H	L	L
**Lifestyle improvement**							
Van Drongelen, A[30] 2014	L	U	H	H	H	H	H
Van het Reve, E[40] 2014	U	U	U	U	H	H	U
**Diabetes management**							
Kirwan, M[41] 2013	H	L	H	H	H	L	L
**Sun protection**							
Buller, D.B[11] 2015	U	U	H	H	L	L	L

Multimedia Appendix 4: Study Quality Assessment

**Hypertension management**							
Moore, J. O[42] 2014	U	L	U	U	L	L	H
**Cardiac rehabilitation**							
Varnfield, M[44] 2014	L	L	H	H	H	H	H
**Smoking cessation**							
Bricker, J. B[45] 2014	L	U	L	L	L	L	H
**Family planning**							
Gilliam ML[9] 2014	L	L	H	L	L	L	L
**Pain management**							
Irvine,A B[10] 2015	U	U	H	H	L	L	L

L: low risk of bias
H: High risk of bias
U: unclear risk of bias
